# Inflammation and cell-to-cell communication, two related aspects in frailty

**DOI:** 10.1186/s12979-022-00306-8

**Published:** 2022-10-26

**Authors:** Orietta Pansarasa, Maria Chiara Mimmi, Annalisa Davin, Marta Giannini, Antonio Guaita, Cristina Cereda

**Affiliations:** 1 IRCCS Mondino Foundation, Via Mondino 2, 27100 Pavia, Italy; 2 Golgi Cenci Foundation, 20081 Abbiategrasso, Milan Italy

**Keywords:** Frailty syndrome, Inflammation, Immune system, Cytokines, Intercellular communication, Extracellular vesicles

## Abstract

**Background:**

Frailty is a complex, multi-dimensional age-related syndrome that increases the susceptibility to adverse health outcomes and poor quality of life. A growing consensus supports the contribution of chronic inflammation and immune system alterations to frailty, however a clear role of such alterations remains to be elucidated. Furthermore, pro- and anti-inflammatory cytokines together with other signaling molecules might spread from activated cells to the adjacent ones through extracellular vesicles (EVs), which have also a role in cellular aging. The aim of the present research was to investigate if EVs play a role in the immune function in frailty.

**Results:**

In 219 older adults aged 76–78 years, selected from the InveCe.Ab study (Abbiategrasso, Italy), we investigated inflammation and EVs-mediated intercellular communication. C-reactive protein (CRP) and pro- (IL-1β, IL-2, IL-6, IL-8, IL-12 p70, TNFα and IFNγ) and anti- (IL-4, IL-10, IL-13) inflammatory cytokines were evaluated on plasma of Frail and non-Frail subjects. We reported a significant increase in CRP, interleukin-1β and -6 (IL-1β, IL-6) and tumor necrosis factor alpha (TNFα) plasma levels in frailty. In female Fr subjects, we also reported an increase in interferon‐gamma (IFN‐γ) and, surprisingly, in IL-13, an anti-inflammatory cytokine, whose increase seems to oppose the inflammaging theory.

An inflammatory panel (toll-like receptors 2 and 4 (TLR2 and TLR4), tumor necrosis factor receptors TNFRec5/CD 40 and TNFRec1B/CD120B) and a panel including receptors involved in cellular senescence (insulin-like growth factor 1 receptor (CD221) and interleukin 6 receptor (IL-6R)) were indeed analysed in plasma isolated large EVs (lEVs) from Frail (*n* = 20) and non-Frail (*n* = 20) subjects. In lEVs isolated from plasma of Frail subjects we reported an increase in TLR2 and TLR4, TNFRec5/CD 40 and TNFRec1B/CD120B, suggesting a chronic state of inflammation. In addition, CD221 and IL-6R increases in lEVs of Frail individuals.

**Conclusions:**

To conclude, the pro-inflammatory status, notably the increase in circulating cytokines is pivotal to understand the potential mechanisms underlying the frailty syndrome. Moreover, cytokines release from EVs, mainly the large ones, into the extracellular space suggest their contribution to the formation of a pro-inflammatory and pro-senescent microenvironment that, in turn, can contribute to frailty.

**Supplementary Information:**

The online version contains supplementary material available at 10.1186/s12979-022-00306-8.

## Introduction

The increase in life expectancy and the progressive aging of the general population are phenomena occurring worldwide. However, the lack of a parallel increase in health span due to age-related diseases is substantially responsible for the growing of frailty. Frailty is a multidimensional geriatric syndrome, with great health and socio-economic implications. Frailty is characterized by the decline of the physiological functions that leads to multisystemic dysregulation, dependency and vulnerability to stressors, which are all associated with a high risk for adverse health outcomes, including increased risk of falls, morbidity and mortality [1]. Frailty is a dynamic condition from a pre-frail status, in which subjects perform well but are at high risk to become frail and severe frailty, in which physiological systems deteriorate and death is upcoming. Although there is a growing interest in frailty, its biological cause/s are poorly characterized.

Chronic inflammation, immune activation, dysregulation of the musculoskeletal and endocrine systems, oxidative stress, energy imbalances, mitochondrial dysfunction, and sarcopenia are all key physiological systems that are unbalanced and closely related with frailty [2]. In particular, chronic inflammation and immune system contribute both to frailty [3], however a precise causative role of such alterations remains to be determined. The age-related decline in the immune function, termed immunosenescence, also includes *inflammaging*. *Inflammaging* is identified by a chronic, systemic, low grade pro-inflammatory state [4, 5] characterized by increased levels of interleukins: 1β (IL-1β), interleukin 6 (IL-6), tumor necrosis factor alpha (TNFα) and the C-reactive protein (CRP); while the levels of interleukin 10 (IL-10), an anti-inflammatory cytokine, mainly decrease [6]. The association between pro-inflammatory cytokines and frailty is quite controversial and open to discussion. On the one hand, pro-inflammatory cytokines can affect frailty directly by favoring the degradation of proteins or indirectly by means their action on some metabolic pathways [7]. On the other hand, Yao and co-authors [2011] highlighted that pro-inflammatory cytokines are not the only predictive marker for the incidence of frailty [8].

Despite recent advances, many aspects of *inflammaging* remain to be clarified, as the role of extracellular vesicles (EVs). EVs, once thought to be cellular trash, are now indicated as mediators of the cell-to-cell communication since their membrane and cytosolic proteins, lipids and genetic material can be transferred between cells both in physiological and pathological conditions [9]. EVs, both microvesicles or large extracellular vesicles (lEVs) and exosomes or small extracellular vesicles (sEVs), are released both in physiological and pathological conditions and are implicated in many cellular processes, such as the immune response and they have been related to inflammatory, autoimmune and infectious diseases [10]. Moreover, evidences suggested that EVs also play a role in cellular aging [11, 12]. Monti and colleagues (2017) stressed that pro- and anti-inflammatory cytokines and other signalling molecules might spread from activated cells to adjacent cells through circulating elements and EVs, mainly microvesicles or lEVs [13].

Therefore, we aim to investigate inflammation and altered intercellular communication, both mechanisms involved in the biology of frailty, to ultimately identify if EVs could play a role in the immune function in frail subjects.

## Results

### The acute-phase inflammatory protein CRP increases with frailty

**Fig. 1:**
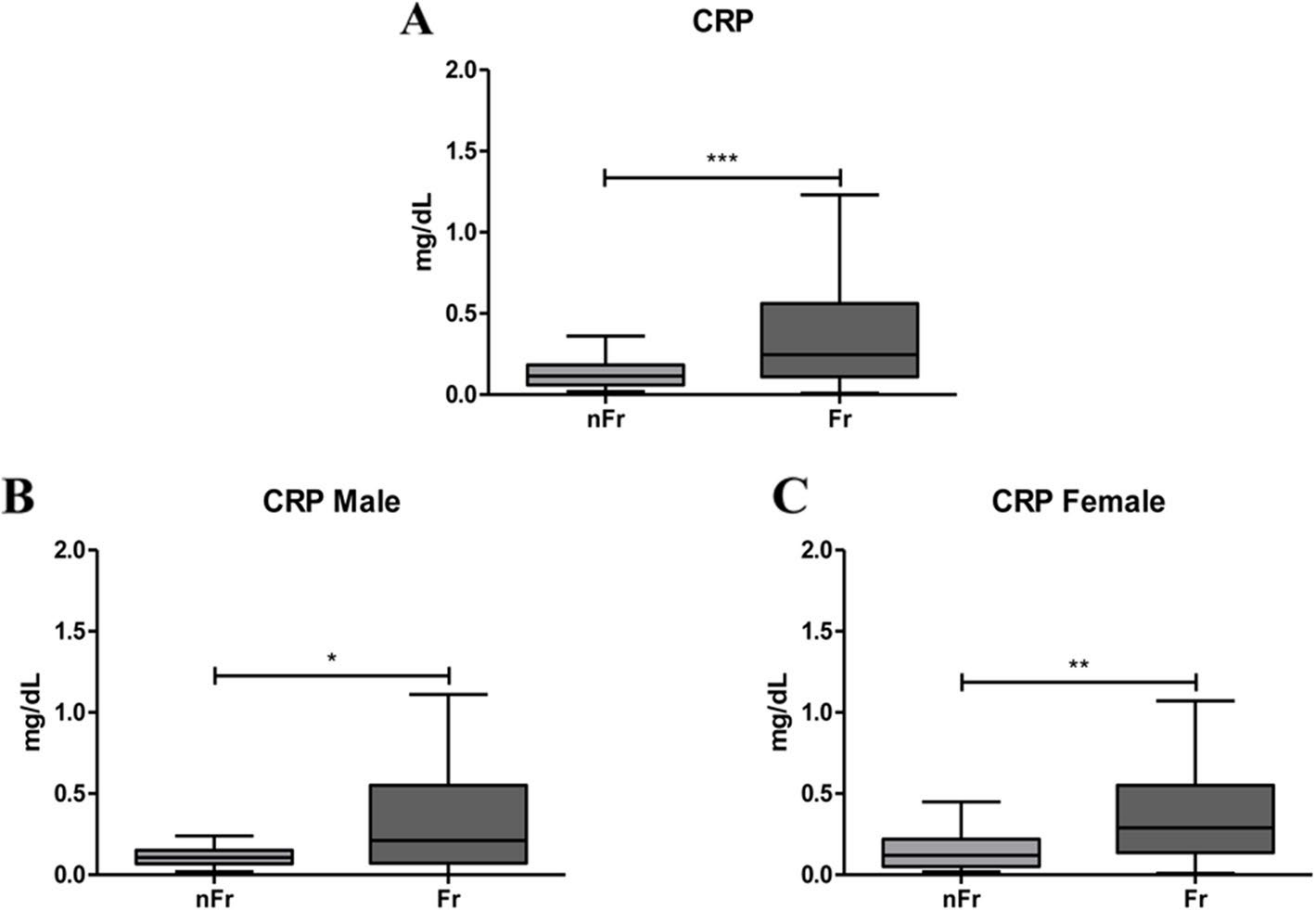
CRP increases with frailty. (**A**) CRP plasma levels are shown to be significantly higher in all Frail (Fr, *n* = 65) compared to nFr (*n* = 64) subjects (***p < 0.001). CRP plasma levels were analyzed according to gender separation; in (**B**) CRP plasma levels increase in Fr male (*n* = 33) compared to nFr male (*n* = 32) subjects (*p < 0.05). In (**C**) are reported data from Fr female (*n* = 32) compared to nFr female (*n* = 32) subjects, a statistically significant increase is observed in Fr vs. nFr subjects (**p < 0.01). Statistical analysis was performed using the Mann–Whitney U test (non-parametric) for comparisons between two groups using GraphPad Prism8 software. A *p* < 0.05 was considered statistically significant

In our cohort of non-Frail (nFr) and Frail (Fr) subjects, we primary aimed to search for the relation between the common inflammatory marker CRP and frailty. We tested plasma CRP concentration in Fr (*n* = 65) and nFr (*n* = 64) subjects and the analysis of the data showed a significant higher concentration in Fr compared to nFr subjects (***p < 0.001) (Fig. 1a). The same result was observed when we analyzed plasma CRP concentration after gender stratification: in both male (Fig. 1b) and female subjects (Fig. 1c) we reported a statistically significant increase in Fr versus nFr (*p < 0.05 and **p < 0.01, respectively).

### IL-1β, IL-6 and TNFα plasma levels significantly increase in plasma of Fr subjects compared to nFr

**Fig. 2:**
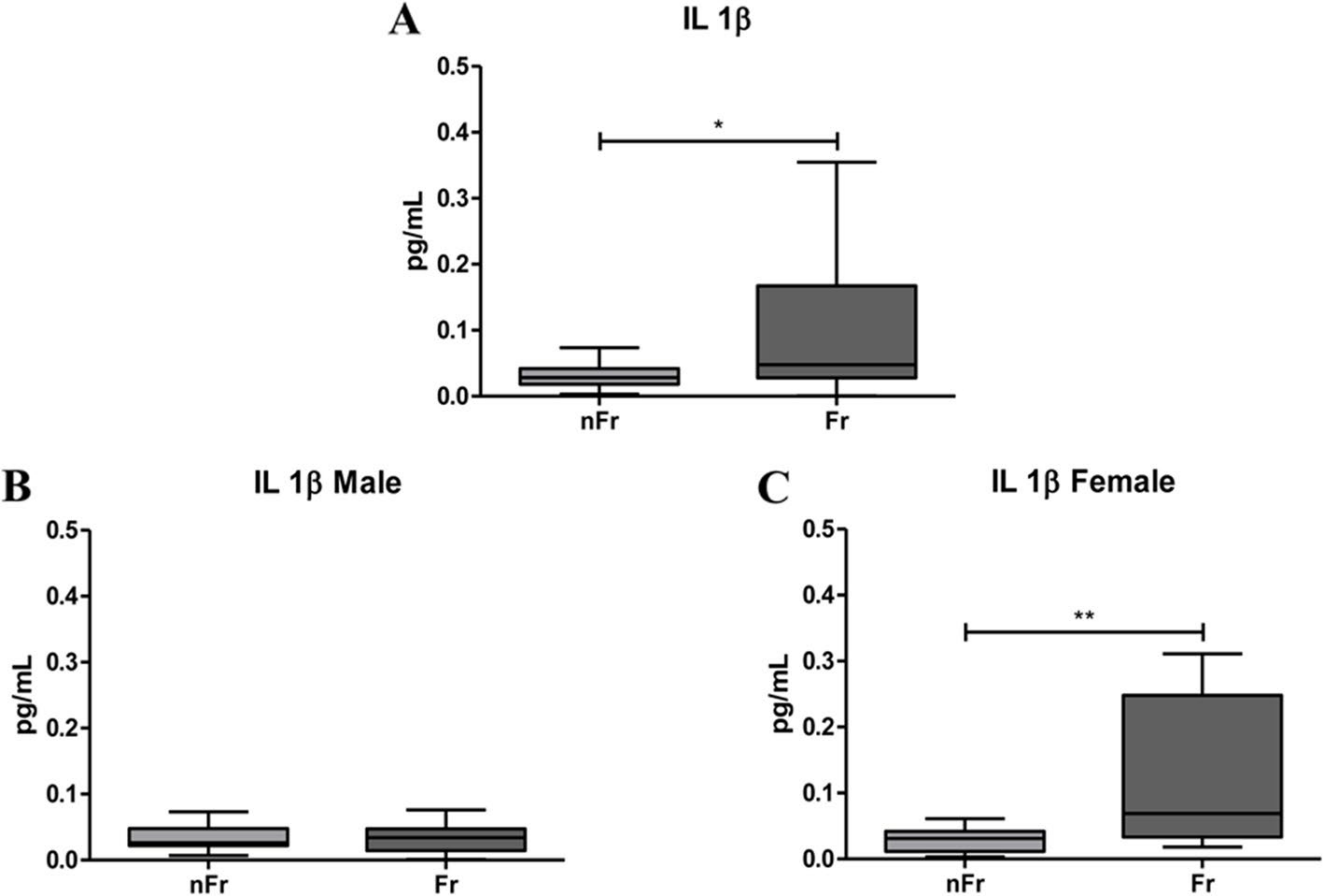
IL-1β plasma levels significantly increase with frailty. (**A**) IL-1β plasma levels are significantly higher in all Frail (Fr, *n* = 65) compared to nFr (*n* = 64) subjects (*p < 0.05). IL-1β plasma levels were analyzed according to gender separation; in (**B**) IL-1β plasma levels in Fr male (*n* = 33) do not change compared to nFr male (*n* = 32) subjects. In (**C**) are reported data from Fr female (*n* = 32) compared to nFr female (*n* = 32) subjects, a statistically significant increase is observed in Fr vs. nFr subjects (**p < 0.01). Statistical analysis was performed using the Mann–Whitney U test (non-parametric) for comparisons between two groups using GraphPad Prism8 software. A *p* < 0.05 was considered statistically significant

**Fig. 3:**
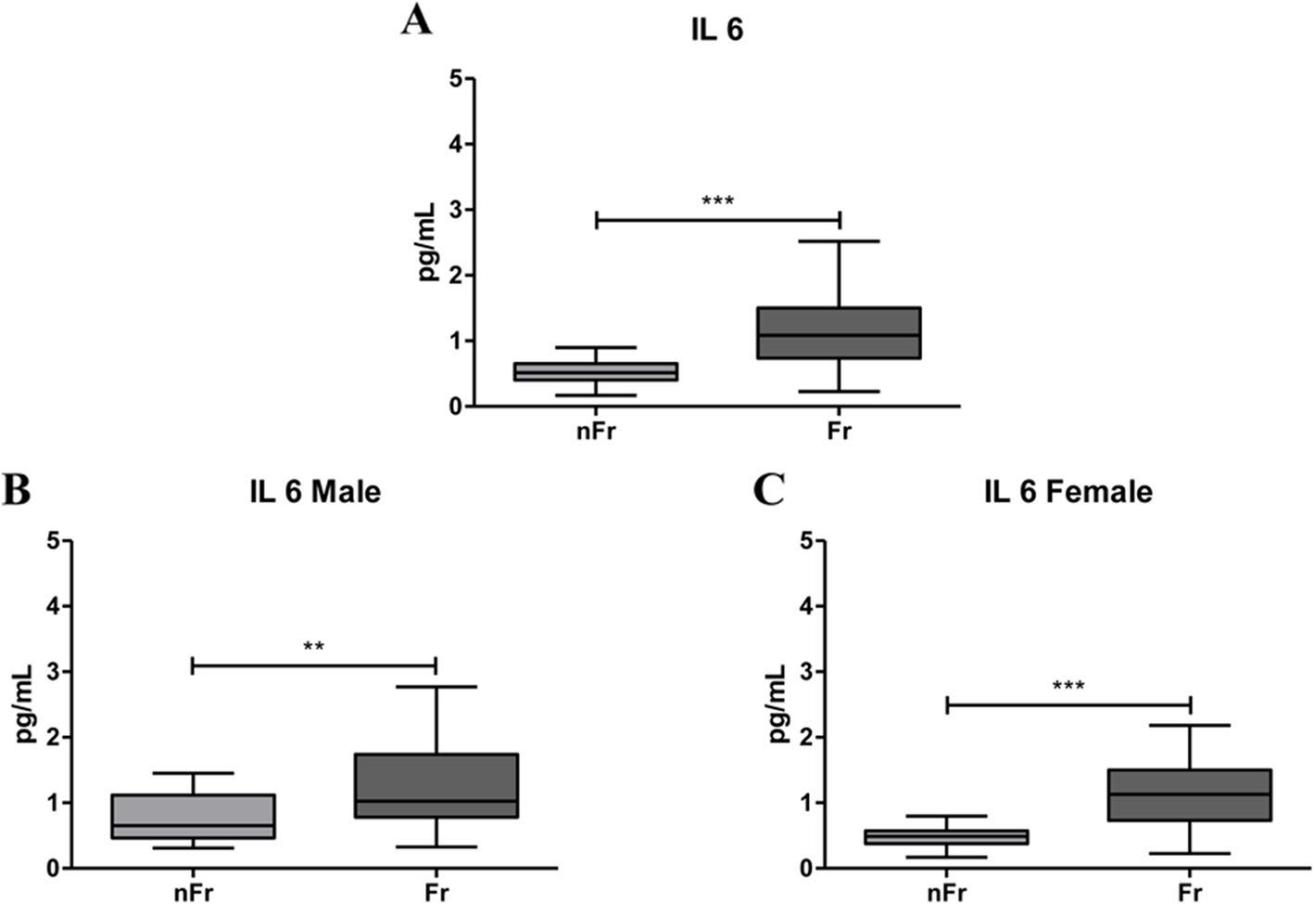
IL-6 plasma levels significantly increase with frailty. (**A**) IL-6 plasma levels are significantly higher in all Fr (*n* = 65) compared to nFr (*n* = 64) subjects (***p < 0.001). IL-6 plasma levels were analyzed according to gender separation; in (**B**) IL-6 plasma levels in Fr male (*n* = 33) significantly increase compared to nFr male (*n* = 32) subjects (**p < 0.01). In (**C**) are reported data from Fr female (*n* = 32) compared to nFr female (*n* = 32) subjects, a statistically significant increase is observed in Fr vs. nFr subjects (***p < 0.001). Statistical analysis was performed using the Mann–Whitney U test (non-parametric) for comparisons between two groups using GraphPad Prism8 software. A *p* < 0.05 was considered statistically significant

**Fig. 4:**
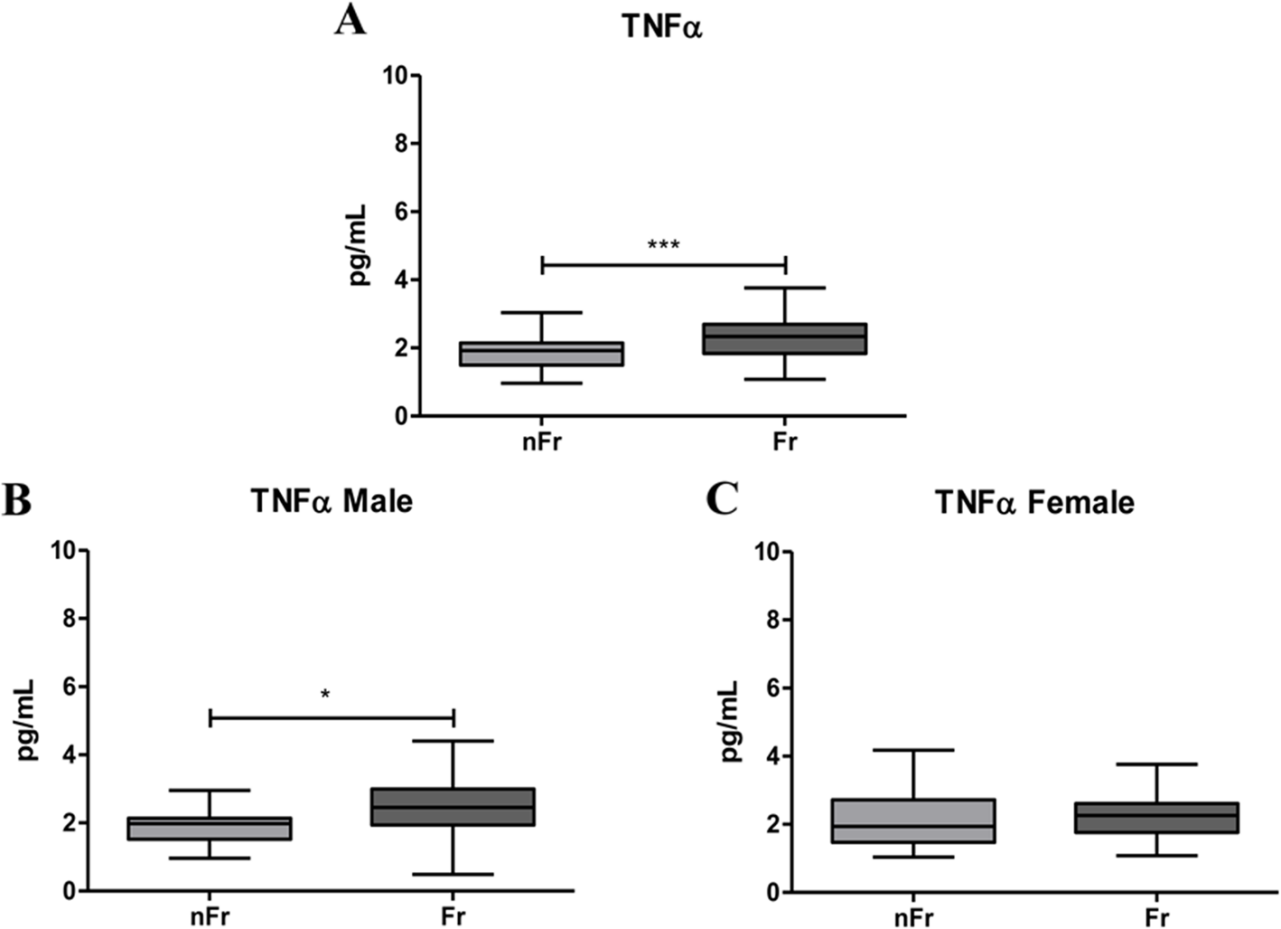
TNFα plasma levels significantly increase with frailty. (**A**) TNFα plasma levels are significantly higher in all Frail (Fr, *n* = 65) compared to nFr (*n* = 64) subjects (***p < 0.001). IL-6 plasma levels were analyzed according to gender separation; in (**B**) TNFα plasma levels in Fr male (*n* = 33) significantly increase compared to nFr male (*n* = 32) subjects (*p < 0.05). In (**C**) are reported data from Fr female (*n* = 32) compared to nFr female (*n* = 32) subjects, no significant differences were reported. Statistical analysis was performed using the Mann–Whitney U test (non-parametric) for comparisons between two groups using GraphPad Prism8 software. A *p* < 0.05 was considered statistically significant

Starting from the evidence that the upregulation of pro-inflammatory cytokines has been associated to frailty, we measured the plasma concentrations of 10 circulating cytokines, by means the Meso Scale Discovery (MSD), in Fr (*n* = 65) and nFr (*n* = 63) subjects (Table 1). When we analyzed all subjects, the Fr group had significant higher levels of *IL-1β* respect to nFr (*p < 0.05) (Fig. 2a-c); while when we grouped samples according to gender an increase in IL-1β in Fr group compared to nFr was observed only in female (**p < 0.01) (Fig. 2c). No differences were observed in male group (Fig. 2b). *IL-6* levels were significantly higher in all Fr subjects compared to nFr (***p < 0.001) and in both female and male Fr subjects compared to nFr (**p < 0.01 and ***p < 0.001, respectively) (Fig. 3a-c). These results confirm the low-grade inflammatory condition of Fr individuals. The levels of *TNFα* were significantly higher in all Fr subjects (***p < 0.001) (Fig. 4a-c) and, when grouping according to gender, we observed plasma TNFα levels significantly higher only in the male Fr (*p < 0.05) than in the nFr individuals (Fig. 4b). According to other groups [14], we reported a significant higher plasma levels of pro-inflammatory cytokines in Fr group compared to nFr. The same tendency was observed when we analyzed male and female separately.

**Table 1:** Results of CRP analysis and cytokine detection by the Meso Scale Discovery (MSD), in Fr (*n* = 65) and nFr (*n* = 63) subjects. The median value, 25^th^ (Q1) and 75^th^ (Q3) percentile values and the *p*-value in the Fr and nFr group were reported

	**nFr**			**Fr**			***p*-value**
	**Median**	**Q1**	**Q3**	**Median**	**Q1**	**Q3**	
**CRP**	0,115	0,06	0,182	0,245	0,11	0,56	< 0.0001
**IFNγ**	4,742	2,667	8,021	5,079	3,358	6,981	0,4331
**IL-10**	0,248	0,177	0,312	0,254	0,169	0,423	0,3323
**IL-12p70**	0,12	0,079	0,164	0,148	0,078	0,201	0,1645
**IL-13**	1,2	0,648	2,498	2,032	0,852	3,213	0,0852
**IL-1β**	0,028	0,018	0,042	0,047	0,028	0,167	0,0108
**IL-2**	0,139	0,088	0,193	0,153	0,099	0,207	0,2116
**IL-4**	0,019	0,007	0,038	0,018	0,01	0,035	0,9706
**IL-6**	0,515	0,401	0,653	1,087	0,734	1,5	< 0.0001
**IL-8**	3,12	2,325	3,728	3,316	2,387	4,914	0,1091
**TNFα**	1,92	1,5	2,144	2,337	1,836	2,693	0,0006

*Fr* Frail, *nFr* non-Frail, *Q1* 25^th^ percentile, *Q3* 75^th^ percentile, *CRP* C-reactive protein, *IFNγ* interferon, *IL-10* interleukin-10, *IL-12p70* interleukin-12p70, *IL-13* interleukin-13, *IL-1β* interleukin-1β, *IL-2* interleukin-2, *IL-4* interleukin-4, *IL-6* interleukin-6, *IL-8* interleukin-8, *TNFα* tumor necrosis factor α

Among the other cytokines we evaluated, only the plasma levels of interferon‐gamma (IFN‐γ) and IL-13 showed statistically significant differences (*p < 0.05 for both) between Fr and nFr subjects in the female group (Supplementary Fig. 1a,b).

### lEVs size and concentration profile do not change in Fr individuals

Starting from the increased inflammatory state observed in the Fr group, we then investigated the involvement of EVs, specifically of lEVs, in frailty. To assess the size profile and the concentration of circulating lEVs, nanoparticle tracking analysis (NTA) was performed. A representative image obtained by NTA is reported in Supplementary Fig. 2a. Size and concentration of lEVs, obtained from plasma of Fr (*n* = 20) and nFr (*n* = 20) subjects, showed no significant differences between the two subject groups (Fr and nFr) (Supplementary Fig. 2b,c). No differences in size and concentration were observed according to gender separation (data not shown).

### The inflammatory markers highlight a chronic state of inflammation in lEVs of Fr subjects

Immune cells communicate with each other by binding to receptors or by delivering vesicles, also containing cytokines. Regarding the inflammatory markers two different panels were employed. The first for T lymphocyte markers (CD3, CD4 and CD8), macrophage markers (CD163) and finally for B and T activated lymphocytes (CD197). For none of these markers, differences were observed in the Fr group compared to nFr (Data not shown).

However, there are considerable body of evidence indicating that circulating EVs increase during inflammatory events and that EVs can expose cytokine receptors to arrest the inflammatory status [12]. Thus, we searched for other inflammatory markers; we measured the toll-like receptors 2 and 4 (TLR2 and TLR4), the tumor necrosis factor receptors TNFRec5/CD 40 (briefness CD40) and TNFRec1B/CD120B (briefness CD120B) in plasma isolated lEVs from Fr and nFr subjects (Supplementary Table 1).

**Fig. 5:**
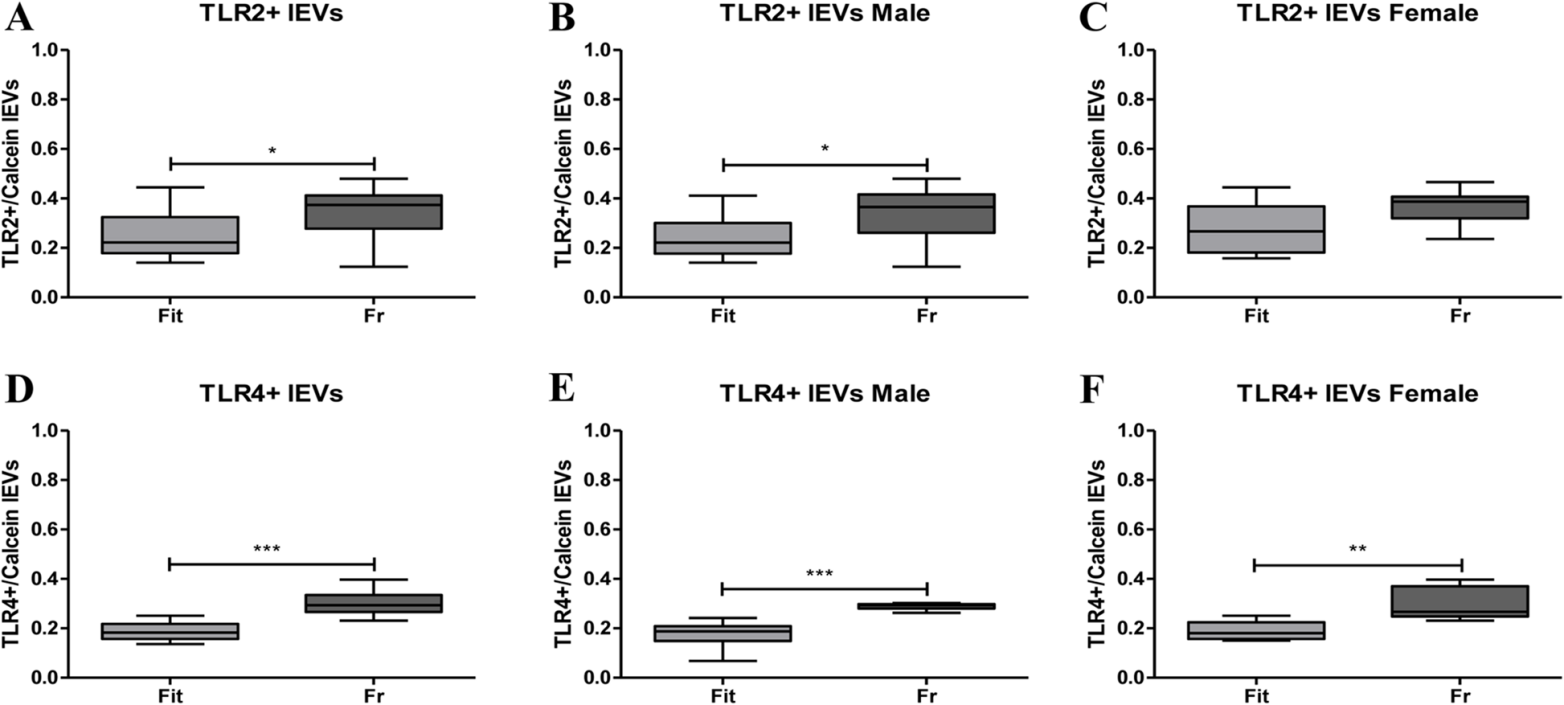
The inflammatory markers highlight an increase in TLR2 + /calcein + lEVs and TLR4 + /calcein + lEVs in Fr subjects. **A** TLR2 + /calcein + lEVs show significantly higher plasma concentration in all Fr (*n* = 20) compared to nFr (*n* = 20) subjects (*p < 0.05). **B** A significant increase (*p < 0.05) was reported in male Fr (*n* = 7) compared to Fit (*n* = 11) subjects. **C** No differences were reported in the female Fr (*n* = 13) versus nFr (*n* = 9) group. **D** TLR4 + /calcein + lEVs show significantly increase higher plasma concentration in all Fr (*n* = 20) compared to nFr (*n* = 20) subjects (***p < 0.001). **E** A significant increase (***p < 0.001) was reported in male Fr (*n* = 7) compared to nFr (*n* = 11) subjects. **F** The female Fr (*n* = 13) group shows a significant increase (**p < 0.01) in TLR4 + /calcein + lEVs versus nFr (*n* = 9). Statistical analysis was performed using the Mann–Whitney U test (non-parametric) for comparisons between two groups using GraphPad Prism8 software. A *p* < 0.05 was considered statistically significant

**Fig. 6:**
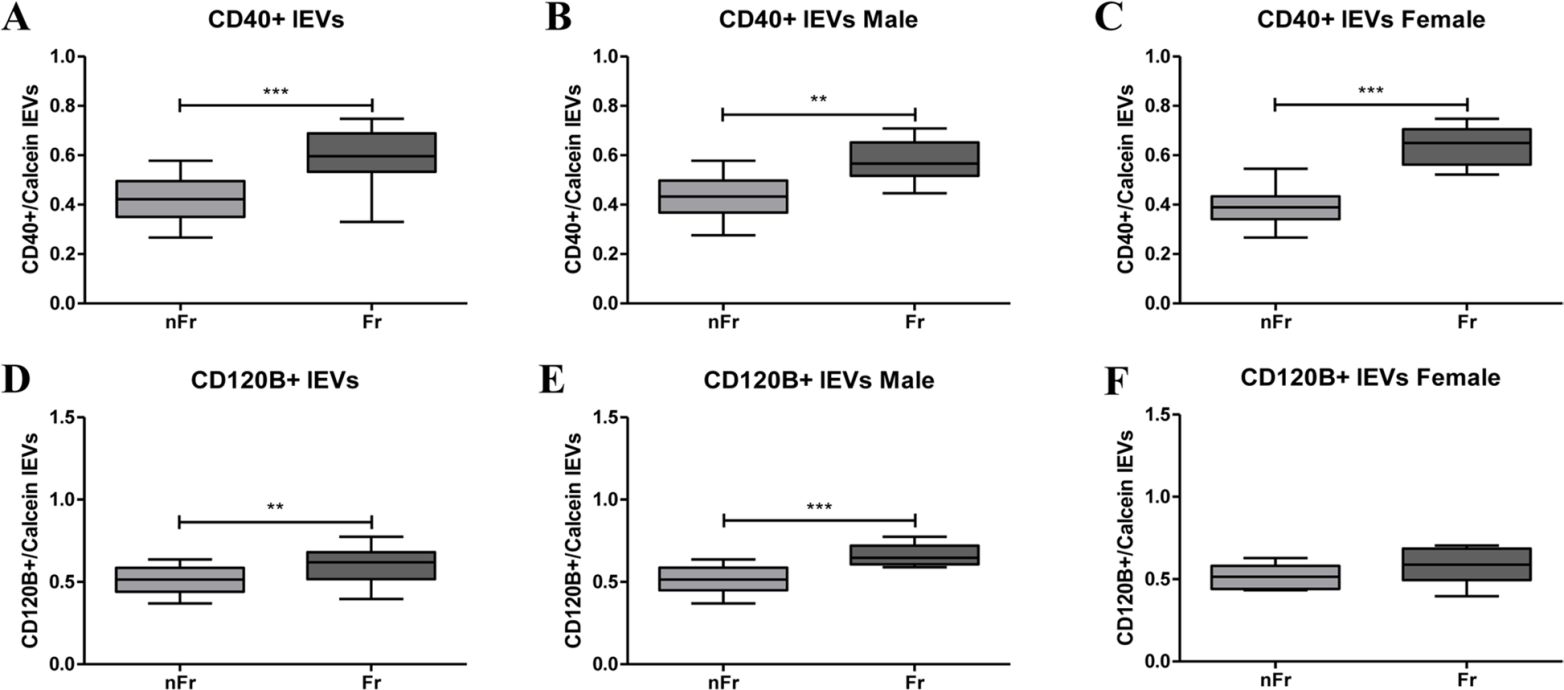
The inflammatory markers highlight an increase in CD40 + /calcein + and CD120 + /calcein + lEVs in Fr subjects. **A** CD40 + /calcein + lEVs show significantly higher plasma concentration in all Fr (*n* = 20) compared to nFr (*n* = 20) subjects (***p < 0.001). **B** A significant increase (**p < 0.01) was reported in male Fr (*n* = 7) compared to nFr (*n* = 11) subjects. **C** The female Fr (*n* = 13) group shows a significant increase (***p < 0.001) in CD40 + /calcein + lEVs versus nFr (*n* = 9). **D** CD120 + /calcein + lEVs show significantly higher plasma concentration in all Fr (*n* = 20) compared to nFr (*n* = 20) subjects (**p < 0.01). **E** A significant increase (***p < 0.001) was reported in male Fr (*n* = 7) compared to nFr (*n* = 11) subjects. **F** No differences were reported in the female Fr (*n* = 13) versus nFr (*n* = 9) group. Statistical analysis was performed using the Mann–Whitney U test (non-parametric) for comparisons between two groups using GraphPad Prism8 software. A *p* < 0.05 was considered statistically significant

*TLR2* + */calcein* + *lEVs* significantly increase (*p < 0.05) in all Fr subjects compared to nFr (Fig. 5a). When subjects were grouped according to gender, we observed a significant increase in male Fr subjects, while no differences were reported in the female group (Fig. 5b,c). Accordingly, also *TLR4* + */calcein* + lEVs significantly enhance (***p < 0.001) in all Fr subjects compared to nFr (Fig. 5d). Moreover, when looking at the differences after gender stratification, we reported a significant increase in TLR4 + /calcein + lEVs in both male (***p < 0.001) and female (**p < 0.01) Fr respect to nFr subjects (Fig. 5e,f). A statistically significant increase was also reported in *CD40* + */calcein* + *lEVs* in all Fr group (***p < 0.001) compared to nFr (Fig. 6a). The significant increase was maintained when grouping by gender (**p < 0.001 for male and ***p < 0.001 for female) (Fig. 6 b,c). Finally, we reported an increase in *CD120B* + */calcein* + *lEVs* in all Fr subjects compared to nFr (Fig. 6d) and also in male Fr subjects (Fig. 6e). No differences were observed in the female Fr group (Fig. 6f).

### Cellular senescence receptors are highly present in lEVs of Fr subjects

EVs modulate not only inflammation, but they probably play a key role in regulating aging and frailty. Two different receptors involved in cellular senescence, i.e. the insulin-like growth factor 1 receptor (CD221) and the interleukin 6 receptor (IL-6R), were analysed (Supplementary Table 1).

**Fig. 7:**
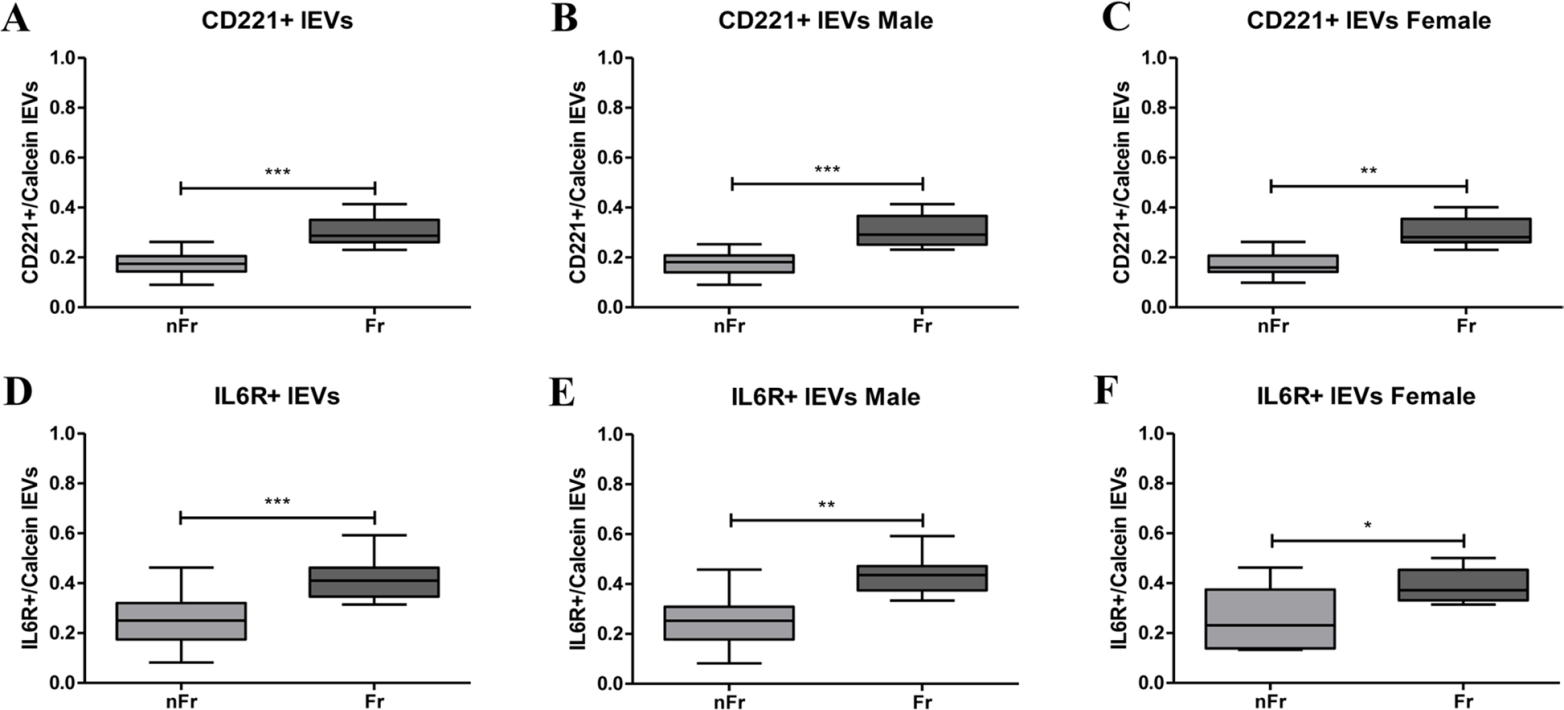
CD221 + /calcein + and IL-6R + /calcein + lEVs increase in Fr subjects. **A** CD221 + /calcein + lEVs show significantly higher plasma concentration in all Fr (*n* = 20) compared to nFr (*n* = 20) subjects (***p < 0.001). **B** A significant increase (***p < 0.001) was reported in male Fr (*n* = 7) compared to nFr (*n* = 11) subjects. **C** The female Fr (*n* = 13) group shows a significant increase (**p < 0.01) in CD221 + /calcein + lEVs versus nFr (*n* = 9). **D** IL-6R + /calcein + lEVs show significantly higher plasma concentration in all Fr (*n* = 20) compared to nFr (*n* = 20) subjects (***p < 0.001). **E** A significant increase (**p < 0.01) was reported in male Fr (*n* = 7) compared to nFr (*n* = 11) subjects. **F** The female Fr (*n* = 13) group shows a significant increase (*p < 0.05) in IL-6R + /calcein + lEVs versus nFr (*n* = 9). Statistical analysis was performed using the Mann–Whitney U test (non-parametric) for comparisons between two groups using GraphPad Prism8 software. A *p* < 0.05 was considered statistically significant

*CD221* + */calcein* + *lEVs* were statistically increased in the all Fr group (***p < 0.001) compared to nFr (Fig. 7a), this tendency was maintained after gender stratification (***p < 0.001 for male and **p < 0.01 for female) (Fig. 7 b,c). *IL6R* + */calcein* + *lEVs* also increase all Fr subjects (Fig. 7d) as well as when we look at male and female Fr individuals separately (Fig. 7e,f).

## Discussion

The present study aimed at identifying EVs, notably lEVs, as new key players of the immune function in frail subjects, by characterizing circulating cytokines and cytokine receptors exposed on lEVs.

We started with the *CRP* evaluation, an acute phase protein, primarily produced and secreted by the liver as a response to inflammatory elements released by T-cells and macrophages [15]. Several studies reported a strong association of CRP plasma levels with frailty [16, 17]. CRP plasma levels were significantly lower in healthy and successfully aged subjects respect to those with age-related diseases or syndromes, including frailty [18]. The authors correlated the increase in CRP concentration with a reduced survival, a poor physical performance and a decline in cognitive performance [18]. In longitudinal studies, the association between CRP and frailty is less definite; Soysal et al. (2016) found that CRP levels were associated with frailty cross-sectionally, but not in longitudinal studies [19]. A longitudinal study has indeed found an association between serum CRP at baseline and incident frailty [20]; while in 2013 Gale and collaborators found this association only in women [21]. All of these studies were completed in similarly aged cohorts (60–70 years) and all but one [22] used the frailty phenotype approach. In our cohort, we evidenced an increase in CRP plasma concentration in Fr subjects. This increase is further emphasized when we grouped our subjects according to gender. Thus our data strengthen the role of CRP as a biomarker in subjects experiencing symptoms of frailty. However, the identification of the CRP value as a predictive biomarker of frailty will require further longitudinal studies.

CRP results strongly suggested a relation between frailty and inflammation, named as immunosenescence. Immunosenescence is usually characterized by an increase in pro-inflammatory cytokines and a reduction in the anti-inflammatory ones which, in turn, generate a chronic low-grade inflammatory state. To investigate cytokine role in frailty we measured a panel of ten different cytokines including pro- (IL-1β, IL-2, IL-6, IL-8, IL-12 p70, TNFα and IFNγ) and anti- (IL-4, IL-10, IL-13) inflammatory cytokines. On the whole, our results showed positive correlation between frailty and IL-1β, IL-6 and TNFα plasma levels. Specifically, we reported a significant increase in *IL-1β* plasma levels in all Fr subjects. The same increase persists in Fr female individuals compare to nFr, while no differences were reported in male. The majority of the studies analyzing the concentration of *IL-1β* in plasma reported no detectable association with frailty [23, 24], only Leng and colleagues (2004) showed a not significant trend of decrease in frail respect to not frail subjects [25]. Data about a strong association between IL-1β and frailty are limited and mainly related to the increased risk of cognitive decline and development of Alzheimer’s disease in the aged individuals [26] or with depression in aged individuals [27]. Concomitantly, we reported a significant increase in IL-6 plasma levels in all Fr individuals. IL-6 plasma levels increase also in the female and male Fr group compared to nFr. Our results strongly agree with data in literature indicating the IL-6 levels increase with age and in frail individuals [28] and in subjects with chronic disease, where it is associated with mortality [18]. These evidences further confirm the contribution of *inflammaging* in the pathophysiology of frailty. Finally, we reported that TNFα plasma levels are elevated in the Fr group and, when male and female are separately considered, the increase was described only in male group. TNFα is another key player in the immune response as it is a pro-inflammatory cytokine, which enhances with age and is associated with age-related disease [29]. It is well known that TNFα is a pro-inflammatory mediator that can be useful when it operates locally, but that can be detrimental when released systemically as in frail individuals. According to literature [30], in our cohort the inflammatory response seems to be different in Fr compared to nFr subjects when we sub-grouped according to gender, In the female Fr group we reported higher plasma levels of CRP, IL-1β and IL-6 respect to the Fit group, while male Fr subjects showed higher levels of CRP, IL-6 and TNFα respect to nFr. These gender-related differences agree with literature, indeed male sex and advanced age was previously associated with increased levels of TNFα together with prostaglandins in response to inflammatory stimuli [31]. The higher levels of plasma IL-1β reported only in female Fr and not in male Fr subjects support the evidence of the crucial role of chronic inflammation in the pathophysiology of frailty in female. Taken together our data point the robust involvement of *inflammaging* in the frailty syndrome and suggest that the presence of circulating cytokines can help the identification of frailty. Surprisingly, we reported an increase in IL-13, an anti-inflammatory cytokine, in female Fr subjects which seems to oppose the *inflammaging* theory. Contradictory data emerge from literature. Palmeri and colleagues (2012) highlight a significant increase in IL-13 levels in nonagenarians versus controls, thus suggesting the importance of anti-inflammatory cytokines in very advanced ages [32]. More recently, Serre-Miranda and colleagues (2020) associated high plasma levels of IL-13 with “Poor” cognitive performance [33], thus pointing out the possibility of a cognitive impairment in Fr female. However, more studies are needed to deeply explore less studied immune molecules like IL-13 and others.

However, many aspects of *inflammaging* remain to be clarified, i.e. the involvement of EVs, both large (lEVs) and small (sEVs). In the present study, we focused on the role of lEVs and pro-inflammatory and pro-senescent hallmarks in frailty. Our data showed that lEVs size and concentration are not affected by frailty where the low-grade pro-inflammatory status observed in our Fr subjects seems to be not sufficient to alter lEVs size and concentration. We employed two different panels related to inflammation to study specific changes that occur to lEVs and to elucidate their role in frailty. No changes in T cell markers (CD3, CD4 and CD8), in macrophage marker (CD 163) and in B and T activated lymphocyets (CD197) lEVs are found in Fr individuals compared to Fit (data not shown). On the other hand, TLR2 and 4 representative of the innate immunity, CD40 involved in adaptive immunity and CD120b (TNF Receptor II) involved in systemic inflammation and capable of stimulating the acute phase reaction are significantly more expressed in lEVs of Fr subjects than in nFr. These results represent a first and clear report of the involvement of lEVs in the activation of pro-inflammatory pathways in the Fr individuals.

Moreover, we tested whether lEVs also express different receptors involved in cellular senescence, notably the IGF1 receptor (CD221) and the IL6 receptor (IL-6R). We reported a significant increase in lEVs that were positive to both IGF1 receptor and IL-6R in Fr individuals compared to nFr. These evidences suggest that IGF1 could be designated as a new potential biomarker of frailty, although further studies are needed in this direction.

Moreover, positivity for the IL-6R and the concomitant increase in circulating IL6 suggest a possible crucial role of EVs in the delivery of cytokines through extracellular release and absorption. Specifically, a potential hypothesis focuses on the release of cytokines from the vesicles directly into the extracellular space in which the cytokines themselves could exert their effect. We do not have data about the EVs cargo, in particular about cytokine cargo, in Fr subjects. To date also in the literature there is a lack in this area of research; in sight of this, an in-depth investigation of EVs cargo could be relevant to further understand their role in a complex, multi-dimensional geriatric syndrome as frailty.

## Conclusions

To conclude, our data strongly suggest that the pro-inflammatory status, specifically the presence of circulating cytokines observed in Fr subjects is crucial to define the frailty syndrome. Moreover, we highlight the role of cell-to-cell communication as a central mechanism in frailty. In particular, EVs and especially lEVs may contribute to the creation of a pro-inflammatory and pro-senescent microenvironment that acts as one of the major contributors to frailty.

## Materials and methods

### Frailty

Frailty was estimated using a frailty index (FI), calculated on the basis of accumulation of deficits, a method widely used in older adults [34]. In our protocol, the FI was based on a list of 32 health variables. Each of these 32 items was assigned a score of 0 or 1, where 0 = absence and 1 = presence of the deficit. Only one item (body mass index) was coded in three classes. Each participant’s scores were then combined in a single index by dividing their number of deficits (ranging from 0 to 32) by the total number considered (32). A final index of 1 corresponded to maximum frailty. On the basis of the FI values obtained, the participants were categorized into three groups: non-Frail (FI ≤ 0.08), PreFrail (FI between 0.08 and 0.25), and Frail (FI ≥ 0.25), as reported in the literature [35]. For the present analysis only non-Frail and Frail subjects were included.

### Study design and participants

This study was conducted in the framework of InveCe.Ab (Invecchiamento Cerebrale in Abbiategrasso), an ongoing longitudinal population-based study (ClinicalTrials.gov, NCT01345110). The study design and the methods have been detailed in Guaita et al*.* (2013) [36]. The study procedures were in accordance with the principles of the Declaration of Helsinki of 1964 and amendments. The study protocol was reviewed and approved by the Ethics Committee of the University of Pavia on October 6th, 2009 (Committee report 3/2009). All the participants gave their written informed consent to the study.

All the InvecCe.Ab participants underwent a multidimensional evaluation to assess social, clinical, and neuropsychological aspects. Sociodemographic variables were gender, age (calculated at each medical examination), and education (number of school years completed). All the information were obtained by means of a questionnaire administered by trained interviewers. They were also asked to provide a blood sample for biochemical and biological analyses and DNA extraction.

A baseline assessment of the InveCe.Ab study was carried out in 2010, enrolling 1321 individuals, and continued with follow-up after 2, 4, and 8 years. The present study relies on 4-year (2014, 1010 participants) examinations. Table 2 reports the descriptive data and number of subjects used for each analysis. The percentage of female subjects is indicated.

**Table 2:** Descriptive data and number of subjects used for the analysis

	***Total***	***non-Frail***	***Frail***	***P***
***CRP plasma analysis***				
N	129	64	65	
Age(years)	76,67 (± 1,38)	76.70 (± 1.34)	76.65 (± 1.42)	0.815
Education (years)	7.06 (± 3.59)	7.64 (± 3.89)	6.49 (± 3.19)	0.069
Frailty Index (0–1)	0.24 (± 0.18)	0.042 (± 0.03)	0.3635 (± 0.11)	< 0.001
Female %	49.6	50	49.2	0.930
***Meso Scale Discoveryfor cytokines analysis***				
N	128	63	65	
Age(years)	76,67 (± 1,37)	76,70 (± 1,38)	76,65 (± 1,42)	0.830
Education (years)	7,09 (± 3,55)	7,71 (± 3,82)	6,49 (± 3,19)	0.520
Frailty Index (0–1)	0.21 (± 0.18)	0.04 (± 0.03)	0.36 (± 0.11)	< 0.001
Female %	50	50.8	49.2	0.860
***lEVs analysis***				
N	40	20	20	
Age(years)	76.60 (± 1.35)	76,50 (± 1,28)	76,70 (± 1,45)	0.647
Education (years)	7.08 (± 3.93)	7,60 (± 4,41)	6,55 (± 3,41)	0.406
Frailty Index (0–1)	0.20 (± 0.21)	0.01 (± 0.007)	0.3941 (± 0.10)	< 0.001
Female %	45	35	55	0.341^a^

^a^Fisher exact test

The Mean values (± Standard Deviation) of the descriptive data of the samples are reported both for the whole sample (Total) and for non-Frail (nFr) and Frail (Fr) subgroups, while as a percentage for the female gender. The comparison between the means was made with the Student's *t* for independent groups, whereas the Chi-square, or the Fisher's exact test, when indicated, was adopted for the percentage. “*P*” column shows the alpha probability. “N” sample size. “lEVs” large extracellular vesicles. The Frailty Index varies between zero and one: subjects ≤ 0.08 were considered nFr, subjects ≥ 0.25 were considered Fr.

### Isolation of plasma and plasma derived large extracellular vesicles (lEVs)

Venous blood (15 mL) was collected in sodium citrate tubes from all nFr and Fr subjects and processed as previously described [37, 38]. Briefly, within 1 h venous blood was centrifuged at 1000 × g for 15 min to separate plasma, followed by an additional centrifugation at 1600 × g for 20 min to remove platelets. The obtained plasma was aliquoted and 500 µL was stored at -80 °C and processed for CRP plasma level analysis and for Meso Scale Discovery for cytokines analysis.

The remaining plasma was the centrifuged at 20,000 × g for 1 h with Centrifuge 5427 R (Eppendorf, Italy). The pellet was washed with 0.22 μm filtered 1 × PBS and centrifuged for 1 h at 20,000 × g. The pellet was then processed for lEVs analysis by means the Nanoparticle-tracking analysis (NTA) [37, 38].

### CRP plasma levels

The C-reactive protein (CRP) concentration was measured using a commercial assay on Cobas 6000 automated analyzer (Roche, Germany). Reference range for CRP was 0–0.5 mg/dL.

### Meso Scale Discovery for cytokines

Proinflammatory Panel 1 (Human) kit, for IL-1β, IL-2, IL-4, IL-6, IL-8, IL-10, IL-12p70, IL-13, IFNγ, TNFα, were purchased from MSD (N05049A-1, Meso Scale Discovery, USA). All the reagents were provided with the MSD kit. Briefly, frozen plasma samples were thawed on ice and centrifuged at 2,000 × g for 3 min to remove particulates and then diluted according to manufactures instructions. Each 96-well plate are pre-coated with one of the anti-cytokine antibodies of interest. The plate was washed and samples, calibrators and controls were added to each well. The plate was incubated for 2 h at room temperature. At the end, each well was washed and the detection antibodies were added. The plate was incubated for 2 h at room temperature, at the end the plate was washed and then the MSD Read Buffer was added to each well and the MSD plates were measured on the MSD Sector Imager 2400 plate reader. The raw data was measured as electrochemiluminescence signal (light) detected by photodetectors and analysed using the Discovery Workbench 3.0 software (MSD). A 4-parameter logistic fit curve was generated for each analyte using the standards and the concentration of each sample calculated.

### Nanoparticle-tracking analysis (NTA) of lEVs

LEVs from nFr and Fr were analyzed by NTA using a NS300 instrument (NanoSight, Amesbury, UK) in order to detect size and concentration of lEVs. For a more accurate detection, samples were diluted with filtered 1 × PBS to the optimal concentration (108–109 particles/mL). After dilution, 1 mL of diluted sample was loaded in the machine and read at a rate of about 30 frames/s. Particle movement videos were recorded 3 times per test and dimension and concentration of lEVs were analyzed by the NTA software (version 2.2, NanoSight) (Supplementary Fig. 2a-c).

### Flow cytometry analysis of lEVs

lEVs pellet was stained with Calcein-AM (56,496 Sigma, Italy) according to the literature [39]. It was then incubated with conjugated primary antibodies as listed in Table 3. Samples were analyzed immediately after labeling, using a BD FACS Canto II with BD FACS Diva software (BD Biosciences, USA). A standardized calibrated-bead strategy (Submicron Bead Calibration Kit (0,2 μm-1 μm), Polysciences Inc., USA) was used to discriminate lEVs from background noise and the absolute number of MVs was calculated using Trucount Absolute Counting Tubes IVD (BD Biosciences, USA) as previously described [40]. Logarithmic amplification was used for all channels and results were referred to the Calcein + lEVs, co-expressing another specific cell lineage marker.

**Table 3:** List of lEVs markers tested by flow cytometry analysis

*Inflammatory Panel*	*Senescent Panel*
BV786 Mouse Anti-Human CD282 (TLR2) BD Biosciences	PE Mouse Anti-Human CD221 (Insulin-like growth factor-I (IGF-I) receptor) BD Biosciences
PE Mouse Anti-Human TLR4 (CD284) BD Biosciences	PerCP/Cyanine5.5 anti-human CD126 (IL-6Rα) BioLegend
PE-Cy™7 Mouse Anti-Human CD40 BD Biosciences	
Alexa Fluor® 647 Rat Anti-Human CD120b BD Biosciences	

### Statistical analysis

All statistical analysis were performed using the GraphPad Prism8 software. Tukey and Box-Plot methods were used to exclude the outliers, while the Mann–Whitney test was used to test null hypothesis. *P* < 0.05 was considered statistically significant.

## Supplementary Information

**Additional file 1:** **Supplementary Fig. 1. **IFNγ plasma levels and IL-13 significantly increase in frailty according to gender separation. (**A**) Plasma levels of IFN‐γ are significantly higher in Fr female (*n*= 32) compared to nFr female (*n*= 32) subjects (*p<0.05). (**B**) Plasma levels of IL-13 are significantly higher in Fr female (*n*=32) compared to nFr female (*n*=32) subjects (*p<0.05).

**Additional file 2:** **Supplementary Fig. 2. **lEVs size and concentration profile does not change in Fr individuals. (**A**) A representative image obtained by NTA is reported. (**B, C**) Size and concentration of lEVs, obtained from plasma of Fr (*n*=20) and nFr (*n*=20) subjects, showed no significant differences between the two subject groups.

**Additional file 3:** **Supplementary Table 1. **Results of TLR2+/calcein+, TLR4+/calcein+, CD40+/calcein+, CD120+/calcein+, analysis in Fr (*n*=65) and nFr (*n*=63) subjects. The median value, 25th (Q1) and 75th (Q3) percentile values and the p-value in the Fr and nFr group were reported .

## Data Availability

The datasets generated and/or analysed during the current study are available after reasonable request to the corresponding author in the Zenodo repository, https://doi.org/10.5281/zenodo.5718535.

## Abbreviations

CD221: Insulin-like growth factor 1 receptor
CRP: C-reactive protein:
EVs: Extracellular vesicles
FI: Frailty index
Fr: Frail
IL-1β: Interleukin-1β
IL-6: Interleukin-6
IL-6R: Interleukin 6 receptor
IL-10: interleukin-10
IL-12p70: interleukin-12p70
IL-13: interleukin-13
IL-2: interleukin-2
IL-4: interleukin-4
IL-8: interleukin-8
IFNγ: interferon γ
lEVs: Large extracellular vesicles
nFr: non-Frail
NTA: Nanoparticle-tracking analysis
sEVs: Small extracellular vesicles
TLR2: Toll-like receptors 2
TLR4: Toll-like receptors 4
TNFRec1B/CD120B: Tumor necrosis factor receptors 1B
TNFRec5/CD 40: Tumor necrosis factor receptors 5
TNFα: Tumor necrosis factor alpha
