# Supplementary figures and images for: Inflammation and cell-to-cell communication, two related aspects in frailty
Source: Immun Ageing. 2022 Oct 26;19:49. doi: 10.1186/s12979-022-00306-8 (PMC9598012; doi:10.1186/s12979-022-00306-8)

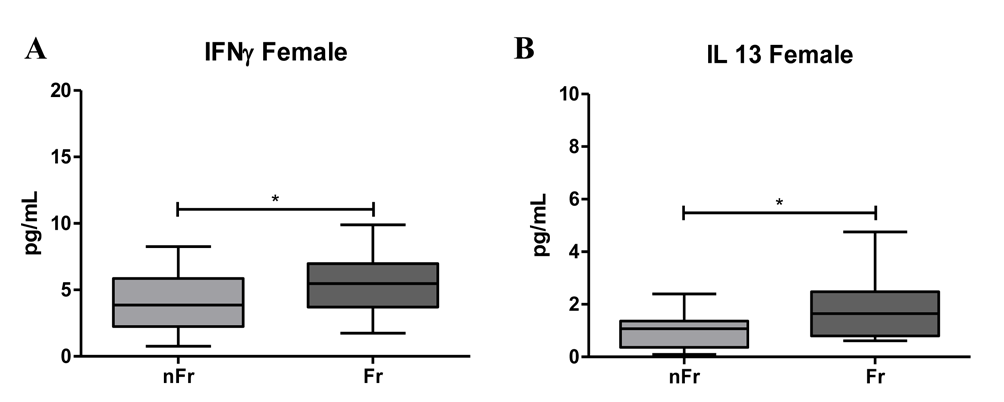

Supplement: Supplementary file 1 — Additional file 1: Supplementary Fig. 1. IFNγ plasma levels and IL-13 significantly increase in frailty according to gender separation. (A) Plasma levels of IFN‐γ are significantly higher in Fr female (n= 32) compared to nFr female (n= 32) subjects (*p<0.05). (B) Plasma levels of IL-13 are significantly higher in Fr female (n=32) compared to nFr female (n=32) subjects (*p<0.05). [file 12979_2022_306_MOESM1_ESM.tif]

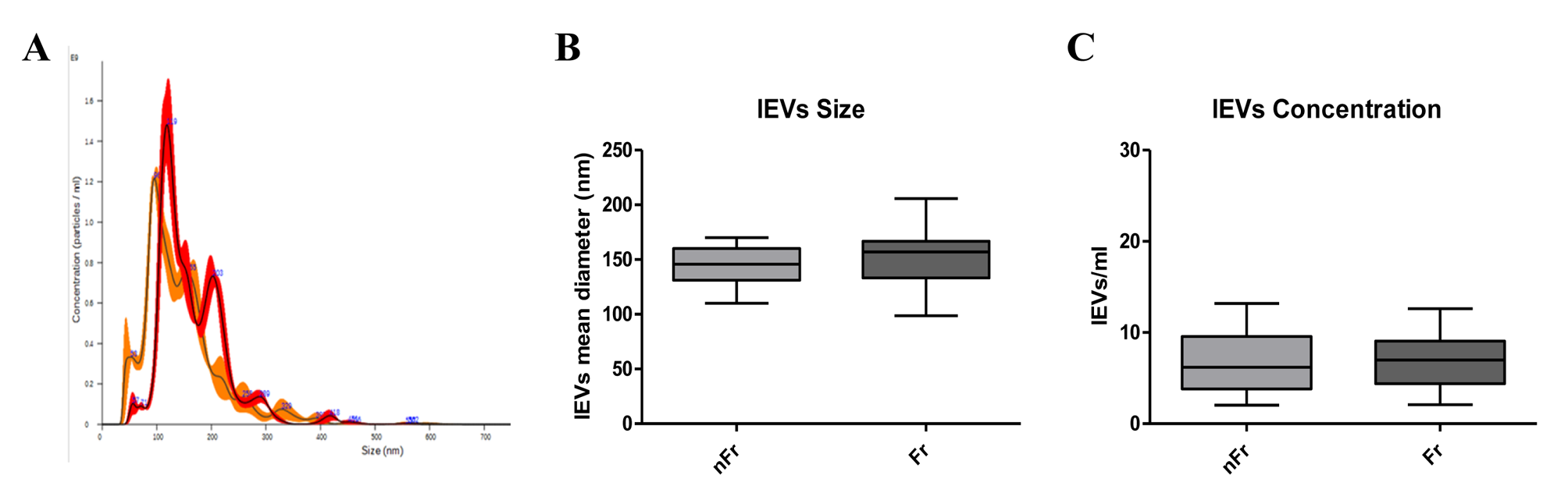

Supplement: Supplementary file 2 — Additional file 2: Supplementary Fig. 2. lEVs size and concentration profile does not change in Fr individuals. (A) A representative image obtained by NTA is reported. (B, C) Size and concentration of lEVs, obtained from plasma of Fr (n=20) and nFr (n=20) subjects, showed no significant differences between the two subject groups. [file 12979_2022_306_MOESM2_ESM.tif]
