# Supplementary material for: Inflammation and cell-to-cell communication, two related aspects in frailty
Source: Immun Ageing. 2022 Oct 26;19:49. doi: 10.1186/s12979-022-00306-8 (PMC9598012; doi:10.1186/s12979-022-00306-8)
Supplement: Supplementary file 3 — Additional file 3: Supplementary Table 1. Results of TLR2+/calcein+, TLR4+/calcein+, CD40+/calcein+, CD120+/calcein+, analysis in Fr (n=65) and nFr (n=63) subjects. The median value, 25th (Q1) and 75th (Q3) percentile values and the p-value in the Fr and nFr group were reported . [file 12979_2022_306_MOESM3_ESM.docx]

**Supplementary Table 1.** Results of TLR2+/calcein+, TLR4+/calcein+, CD40+/calcein+, CD120+/calcein+, analysis in Fr (n=65) and nFr (n=63) subjects. The median value, 25th (Q1) and 75th (Q3) percentile values and the p-value in the Fr and nFr group were reported.

|  | **nFr** | | | **Fr** | | | ***p*-value** |
| --- | --- | --- | --- | --- | --- | --- | --- |
| **lEVs** | **Median** | **Q1** | **Q3** | **Median** | **Q1** | **Q3** |  |
| **TLR2+/calcein+** | 0,222 | 0,1788 | 0,3243 | 0,374 | 0,278 | 0,4125 | 0,0109 |
|  |  |  |  |  |  |  |  |
| **TLR4+/calcein+** | 0,183 | 0,157 | 0,218 | 0,294 | 0,266 | 0,3343 | < 0,0001 |
|  |  |  |  |  |  |  |  |
| **CD40+/calcein+** | 0,422 | 0,3508 | 0,4955 | 0,5965 | 0,533 | 0,6888 | < 0,0001 |
|  |  |  |  |  |  |  |  |
| **CD120B+/calcein+** | 0,5145 | 0,441 | 0,5855 | 0,619 | 0,5173 | 0,68 | 0,0035 |
|  |  |  |  |  |  |  |  |
| **CD221+/calcein+** | 0,175 | 0,1435 | 0,2055 | 0,287 | 0,2613 | 0,3503 | < 0,0001 |
|  |  |  |  |  |  |  |  |
| **IL-6R+/calcein+** | 0,2505 | 0,1743 | 0,3205 | 0,4105 | 0,346 | 0,462 | < 0,0001 |
|  |  |  |  |  |  |  |  |

Fr= Frail; nFr= non-Frail Q1= 25^th^ percentile; Q3= 75^th^ percentile; lEVs= large extracellular vesicles; TLR2= TLR2: Toll-like receptors 2; TLR4= Toll-like receptors 4; CD40= tumor necrosis factor receptors TNFRec5/CD40; CD120B= tumor necrosis factor receptors NFRec1B/CD120B; CD221= insulin growth factor 1 receptor; IL-6R= interleukin 6 receptor.
